# Study on the Infrared and Raman spectra of Ti_3_AlB_2_, Zr_3_AlB_2_, Hf_3_AlB_2_, and Ta_3_AlB_2_ by first-principles calculations

**DOI:** 10.1038/s41598-024-65980-8

**Published:** 2024-07-01

**Authors:** Shengzhao Wang, Lanli Chen, Haoshan Hao, Chong Qiao, Jinfan Song, Chaojun Cui, Bin Liu

**Affiliations:** 1 Nanyang Institute of Technology, School of Mathematics and Physics, No. 80 Changjiang Road, Nanyang, 473004 Henan People’s Republic of China; 2 Henan Province New Optoelectronic and Storage Materials Engineering Technology Research Center, No. 80 Changjiang Road, Nanyang, 473004 Henan People’s Republic of China; 3 College of Mathematics and Physics, Anyang Institute of Technology, Huanghe Avenue, Anyang, 455000 Henan People’s Republic of China

**Keywords:** MAB phase, First-principles, Infrared spectra, Raman spectra, Condensed-matter physics, Structural materials, Theory and computation, Materials science, Physics, Electronics, photonics and device physics

## Abstract

In this paper, the crystal geometry, electronic structure, lattice vibration, Infrared and Raman spectra of ternary layered borides M_3_AlB_2_ (M = Ti, Zr, Hf, Ta) are studied by using first principles calculation method based on the density functional theory. The electronic structure of M_3_AlB_2_ indicates that they are all electrical conductors, and the d orbitals of Ti, Zr, Hf, and Ta occupy most of the bottom of the conduction band and most of the top of the valence band. Al and B have lower contributions near their Fermi level. The lightweight and stronger chemical bonds of atom B are important factors that correspond to higher levels of peak positions in the Infrared and Raman spectra. However, the vibration frequencies, phonon density of states, and peak positions of Infrared and Raman spectra are significantly lower because of heavier masses and weaker chemical bonds for M and Al atoms. And, there are 6 Infrared active modes A_2u_ and E_1u_, and 7 Raman active modes, namely A_1g_, E_2g_, and E_1g_ corresponding to different vibration frequencies in M_3_AlB_2_. Furthermore, the Infrared and Raman spectra of M_3_AlB_2_ were obtained respectively, which intuitively provided a reliable Infrared and Raman vibration position and intensity theoretical basis for the experimental study.

## Introduction

It’s interesting to note that MAB phase materials have gained a lot of attention in recent years due to their similar structure to MAX phase^1–7^. These materials possess unique properties such as a nano-layered structure, diverse chemical bond types, high hardness, high elastic modulus, high conductivity, high thermal conductivity, and good resistance to high-temperature oxidation, making them highly suitable for various applications^8–11^. They can be used to manufacture high-temperature furnaces, aerospace spacecraft components, automotive engine components, nuclear reactor materials, and in other fields^12–15^. In addition, it's worth mentioning that the good thermal conductivity exhibited by MAB phase materials makes them highly suitable for the manufacturing of products like electronic components and radiators^4,16^. These materials also exhibit common characteristics of both ceramics and metals, making them a highly researched area in the field of structural ceramics. The unique properties of MAB phase materials generate significant interest in the ternary layered transition metal boride MAB phase, making it a subject of ongoing research^17–20^. At present, the orthorhombic MAB phases Ru_2_ZnB_2_, Ru_3_Al_2_B_2_, Mo_2_AlB_2_, and Cr_4_AlB_2_ have been successfully prepared. And MAlB (M = Ti, Hf, V, Nb, Ta, Cr, Mo, W, Mn, Tc), M_2_AlB_2_ (Sc, Ti, Zr, Hf, V, Cr, Mo, W, Mn, Tc, Fe, Rh, Ni), M_3_Al_2_B_2_ (M = Sc, T, Zr, Hf, Cr, Mn, Tc, Fe, Ru, Ni), M_3_AlB_4_ (M = Sc, Ti, Zr, Hf, V, Nb, Ta, Cr, Mo, W, Mn, Fe), and M_4_AlB_6_ (M = Sc, Ti, Zr, Hf, V, Nb, Ta, Cr, Mo) were successively studied^21–24^.

However, compared to MAX phases, the study of MAB phases is still in its early stages. Numerical simulation and theoretical analysis based on the first-principles method based on the density functional theory can play an important role in understanding and predicting the phase structure and properties of MAB. In order to gain a deeper understanding of the structure and properties of many MAB phase compounds, it is extremely urgent to employ the first-principles method based on density functional theory to study these materials. This approach will enable the rapid screening and theoretical design of materials with promising engineering application prospects^7,25–43^. Currently, there has been research on Ti_3_AlB_2_ material, but there is still a lack of corresponding research on materials with similar structures or chemical formulas, such as Zr_3_AlB_2_, Hf_3_AlB_2_, Ta_3_AlB_2_, and other related materials. Judging from the limited theoretical studies, these material systems have similar electronic band structures, indicating that they are likely to exhibit similar physical properties^44,45^. Among these materials, Ti_3_AlB_2_, Zr_3_AlB_2_, Hf_3_AlB_2_, and Ta_3_AlB_2_ can form a series of layered materials with a similar structure and chemical composition.

Meanwhile, it is crucial to synthesize these materials and accurately determine their structure from experimental standpoint. And the Infrared or Raman spectroscopy is an effective method for distinguishing these similar crystal structures^46^. However, conducting experimental studies on their Infrared or Raman spectra is time-consuming, labor-intensive, and requires a large workload. We use the first-principles calculations to investigate the Infrared and Raman spectra of these four similar materials M_3_AlB_2_, which will provide us a theoretical reference for subsequent experimental work. Because the Infrared and Raman spectroscopy can provide a variety of information for the study of materials, it has been extended to a variety of disciplines and widely used. Nevertheless, the research on their Infrared and Raman spectrum is extremely few. Therefore, it is of great significance to study the Infrared and Raman spectrum and their vibration mode of M_3_AlB_2_.

## Method and computational details

All first-principles calculations were performed by Vienna Ab-initio Simulation Package (VASP) with Plane-wave DFT methodology in this study^47^. The use of different exchange correlation functionals has been demonstrated to have a minor influence on the formation enthalpy^48–51^. And, the electronic exchange–correlation interactions were described by the generalized gradient approximation (GGA) with Perdew Burke Ernzerhof (PBE) functional^52–55^ in the paper.

In the calculation process, a plane wave cut-off energy of 700 eV was utilized. Additionally, integrations in the Brillouin zone were conducted using a 15 × 15 × 2 Monkhorst–Pack as specific k-points, while a K-mesh of 0.03 was chosen throughout the calculation^56^. Energy and force convergences are 1 × 10^–8^ eV and − 1 × 10^–7 ^eV/Å. In calculations, the Density Functional Perturbation Theory (DFPT) approach was employed in both Phonopy^57,58^ and VASP to determine the second-order Interatomic Force Constants (IFCs). DFPT is a method for calculating the response of physical quantities to external fields using linear response theory. It can be used to calculate the force constant matrix. The Phono3py^59,60^ code generates the displacement structure with irreducible representation along the center q-point to produce positive and negative displacement structures. Finally, the dielectric constant is calculated. Only the structure with irreducible representation is selected for dielectric constant calculation to reduce the calculation burden^61,62^. And Phonopy-Spectroscopy software were utilized in the procedures to calculate the IR and Raman spectra^63^. Hessian matrix data and born effective charges also have to be taken into account. Moreover, the focus of this research was primarily on the atom vibration image of the structure's Γ point active Infrared and Raman spectra associated to phonons. Additionally, 3d^2^4s^2^ for Ti, 4d^2^5s^2^ for Zr, 5d^2^6s^2^ for Hf, 5d^3^6s^2^ for Ta, 3s^2^3p^1^ for Al, and 2s^2^2p^1^ for B were considered as valence states in the paper. The software packages Pymatgen and Vaspvis were used in the study^64,65^.

Lattice dynamics has played a significant role in understanding certain physical characteristics of Infrared and Raman spectra. We employ the Phonopy package and VASP to obtain phonon dispersion and phonon density of states corresponding to lattice vibration in the paper. The phonon vibrational frequencies via DFPT method. Phonon frequencies and eigenvectors are computed in the harmonic limit using the second-order force constant matrix *Φ*_*αβ*_(*jl*, *j*′*l*′), the elements of which are the change in force in the *α* Cartesian direction acting on atom *j* in unit cell *l*, in response to the displacement of atom *j′* in unit cell *l*′ in the *β* direction. The phonon frequencies are the eigenvalues of the dynamical matrix at a given wavevector ***q***:

1 $${D}_{\alpha \beta }^{jj{^{\prime}}}\left(\mathbf{q}\right)=\frac{1}{\sqrt{{m}_{j}{m}_{j}{^{\prime}}}}{\sum }_{l{^{\prime}}}{\Phi }_{\alpha \beta }\left(jl,j{^{\prime}}l{^{\prime}}\right)\text{exp}\left[\begin{array}{c}i{\varvec{q}}\cdot \left(\mathbf{r}\left({j}^{{{\prime}}},{l}^{{{\prime}}}\right)-{\varvec{r}}\left(j,l\right)\right)\end{array}\right]$$

*m*_*j*_ refer to the atomic masses. The long-rang Coulomb interactions lead to non-analytic corrections in the limit q → 0, which cause the splitting of the longitudinal optic (LO) and transverse optic (TO) modes^66^:

2 $${D}_{\alpha \beta }^{{jj}^{{{\prime}}}}\left(q\to 0\right)={D}_{\alpha \beta }^{{jj}^{{{\prime}}}}\left(q=0\right)+\frac{1}{\sqrt{{m}_{j}{m}_{{jj}^{{{\prime}}}}}}\frac{4\pi }{{\Omega }_{0}}\frac{\left[\sum_{\gamma }{q}_{\gamma }{Z}_{\gamma \alpha }^{j}\right]\left[\sum_{\gamma {\prime}}{q}_{\gamma {\prime}}{Z}_{{\gamma }{\prime}\beta }^{{\text{j}}^{{{\prime}}}}\right] }{\sum_{\alpha \beta }{{q}_{\alpha }\varepsilon }_{\alpha \beta }^{\infty }{q}_{\beta }}$$

${Z}_{i,\alpha \beta }^{*}$ is the derivative of the cell polarization along the Cartesian direction *(x, y, z) α* with respect to the atomic displacement of atom *i* along *β* and defined as

3 $${Z}_{i,\alpha \beta }^{*}=\frac{\Omega }{e}\frac{\partial {P}_{\alpha }}{\partial {r}_{i,\beta }}$$

The dynamical charges Z* are called “Born effective-charge tensors”, and can be calculated using perturbation theory. The calculations have been performed by using the LESPILON = True keyword of VASP. *r* is the displacement of the atom *i*. *Ω* is the volume of the cell and *e* is the electronic charge^67^. The Z^*^ tensors were computed using DFPT as implemented in VASP. *Ω* represents the volume of the primitive unit cell, and *e* stand for the elementary charge constant. Within the dipole approximation, the Infrared intensity of an eigenmode *s* can be expressed as the square of the Born effective-charge and eignevector ${X}_{\beta }\left(s,j\right)$ as:

4 $${I}_{IR}\left(s\right)=\sum_{\alpha }{\left|\sum_{j}\sum_{\beta }{Z}_{\alpha \beta }^{*}{X}_{\beta }\left(s,j\right)\right|}^{2}$$

${X}_{\beta }\left(s,j\right)$ represents the normalized vibrational eigenvector of the *j*th phonon mode of the *s*th atom in the unit cell. Raman activity tensors of an eigenmode are evaluated by taking the derivative of the high-frequency macroscopic dielectric constant *ε*^∞^ with respect to the normal mode amplitude *Q(s)* using a central difference scheme is:

5 $$\begin{array}{cc}& {I}_{\text{Raman}}\left(s\right)\propto \frac{\partial \alpha }{\partial Q\left(s\right)}\equiv \frac{\partial {\varepsilon }^{\infty }}{\partial Q\left(s\right)}\approx \frac{\Delta {\varepsilon }^{\infty }}{\Delta Q\left(s\right)}\\ & \end{array}$$

6 $${I}_{\text{Raman},{\alpha \beta }}\left(s\right)=\frac{\Omega }{4\pi }\left[-\frac{1}{2}\frac{{\varepsilon }_{{\alpha \beta }}^{\infty }\left(-s\right)}{\Delta Q\left(s\right)}+\frac{1}{2}\frac{{\varepsilon }_{{\alpha \beta }}^{\infty }\left(+s\right)}{\Delta Q\left(s\right)}\right]$$

*ε*^∞^ can be calculated by DFPT method and can be found in the OUTCAR with careful identification. At the same time, LESPILON = True is necessary. Q is the normal-mode coordinate at the Γ-point and is defined by

7 $$u\left(s,j\right)=Q\left(s\right)\frac{{\varvec{w}}\left(s,j\right)}{\sqrt{{m}_{j}}}$$

***u***(*s*, *j*) represents the atomic displacement. Phonopy and Phonopy-Spectroscopy were used to compute phonon frequencies, as well as Raman intensities^68,69^. The Raman intensities are then defined as:

8 $${I}_{\text{Raman}}=45{\left|\frac{1}{3}\left({I}_{11}+{I}_{22}+{I}_{33}\right)\right|}^{2}+\frac{7}{2}\left[{\left({I}_{11}-{I}_{22}\right)}^{2}+{\left({I}_{11}-{I}_{33}\right)}^{2}+{\left({I}_{22}-{I}_{33}\right)}^{2}+6\left({I}_{12}^{2}+{I}_{13}^{2}+{I}_{23}^{2}\right)\right]$$

In Eq. (8), the notation ${I}_{{\alpha \beta }}$ was used in place of ${I}_{\text{Raman},{\alpha \beta }}\left(s\right)$. With *m*_*j*_ in amu, and Z* in *e*, the calculated IR intensities will have units *e*^2^ amu^−1^. With Q in amu^1/2^Å, the elements of the Raman-activity tensor have units of Å^2^ amu^−1/2^, and the units of the scalar Raman intensity. And the square of the Raman activity are Å^4^amu^−1^ at last ^63,70^.

## Results and discussion

### Structure

**Figure 1:**
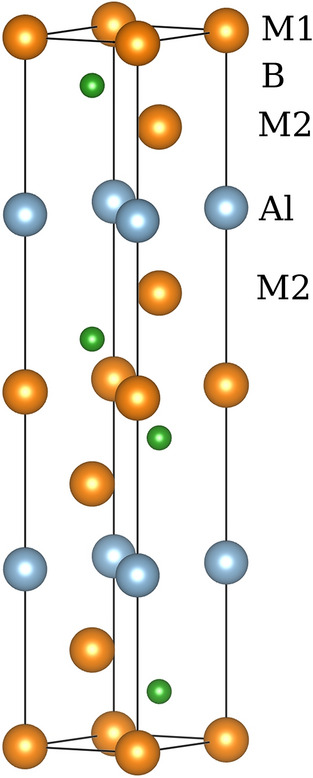
Schematic graphs of M_3_AlB_2_ crystal structure.

In Fig. 1, it is shown that the schematic graphs of crystal structure for M_3_AlB_2_, all of which belong to the P6_3_/mmc (No. 194) space group. Each unit cell contains two primitive cells, resulting in a total of 12 atoms in each conventional unit cell. And the compounds Ti_3_AlB_2_, Zr_3_AlB_2_, Hf_3_AlB_2_, and Ta_3_AlB_2_ can be considered as 312 phases in the M_n+1_AlB_n_ phase structure, which is consistent with references^71,72^. In the M_3_AlB_2_ structure, the Al layer atoms are inserted between the adjacent M layer atoms, and the B atoms fill the octahedral interstitial positions of the former. The Al atom is located at the center of the quadrangular prism, surrounded by the M-layer atoms. Because the space at this position is larger than the octahedral gap, it can accommodate larger atoms such as Al.

### Lattice constant

As shown in Table 1, the lattice constants of Ti_3_AlB_2_, Zr_3_AlB_2_, Hf_3_AlB_2_, and Ta_3_AlB_2_ have been calculated. It can be observed that the calculation results of Ti_3_AlB_2_ are in good agreement with the reference results^72^.

**Table 1:** Lattice constants and c/a ratios of Ti_3_AlB_2_, Zr_3_AlB_2_, Hf_3_AlB_2_, and Ta_3_AlB_2_ (α = β = 90°, γ = 120°).

Materials	a (Å)	c (Å)	c/a	V (Å^3^)
Ti_3_AlB_2_	3.13690	19.54835	6.231742	166.587365
Zr_3_AlB_2_	3.35549	20.51663	6.114347	200.054823
Hf_3_AlB_2_	3.40727	20.69831	6.074749	208.103271
Ta_3_AlB_2_	3.23208	19.85585	6.143366	178.489587

In general, the lattice constants “a” and “c” of the M_3_AlB_2_ phase decrease with the increase in the atomic radius of the transition metal element M, but have little relationship with the number of d electron layers. The lattice constant of the 3d compound is significantly smaller than that of the 4d and 5d compounds, but the difference between the 4d and 5d compounds is minimal.

At the same time, for a series of compounds with a given M_3_AlB_2_ value, the c/a ratio falls within a very narrow range and is less affected by M atoms. We assume that the MAX phase is an interstitial compound, in which A and X atoms fill the interstices between M atoms. In the 312 phase, the c-axis direction comprises 6 layers of M atoms. Without considering the ideal lattice distortion caused by the addition of A and X atoms, the c-axis length should be 6 times that of the a-axis length. That is to say, the c/a ratio of the 312 phase should be 6, and other researchers have also found a similar trend in their investigation^34^. However, in reality, the presence of Al and B atoms inevitably impacts the lattice, causing the actual c/a ratio to deviate slightly from the ideal value. As shown in Table 1, the c/a ratios of Ti_3_AlB_2_, Zr_3_AlB_2_, Hf_3_AlB_2_, and Ta_3_AlB_2_ are 6.231742, 6.114347, 6.074749, and 6.143366, respectively. This aligns with our speculation closely.

### Electronic band structure

**Figure 2:**
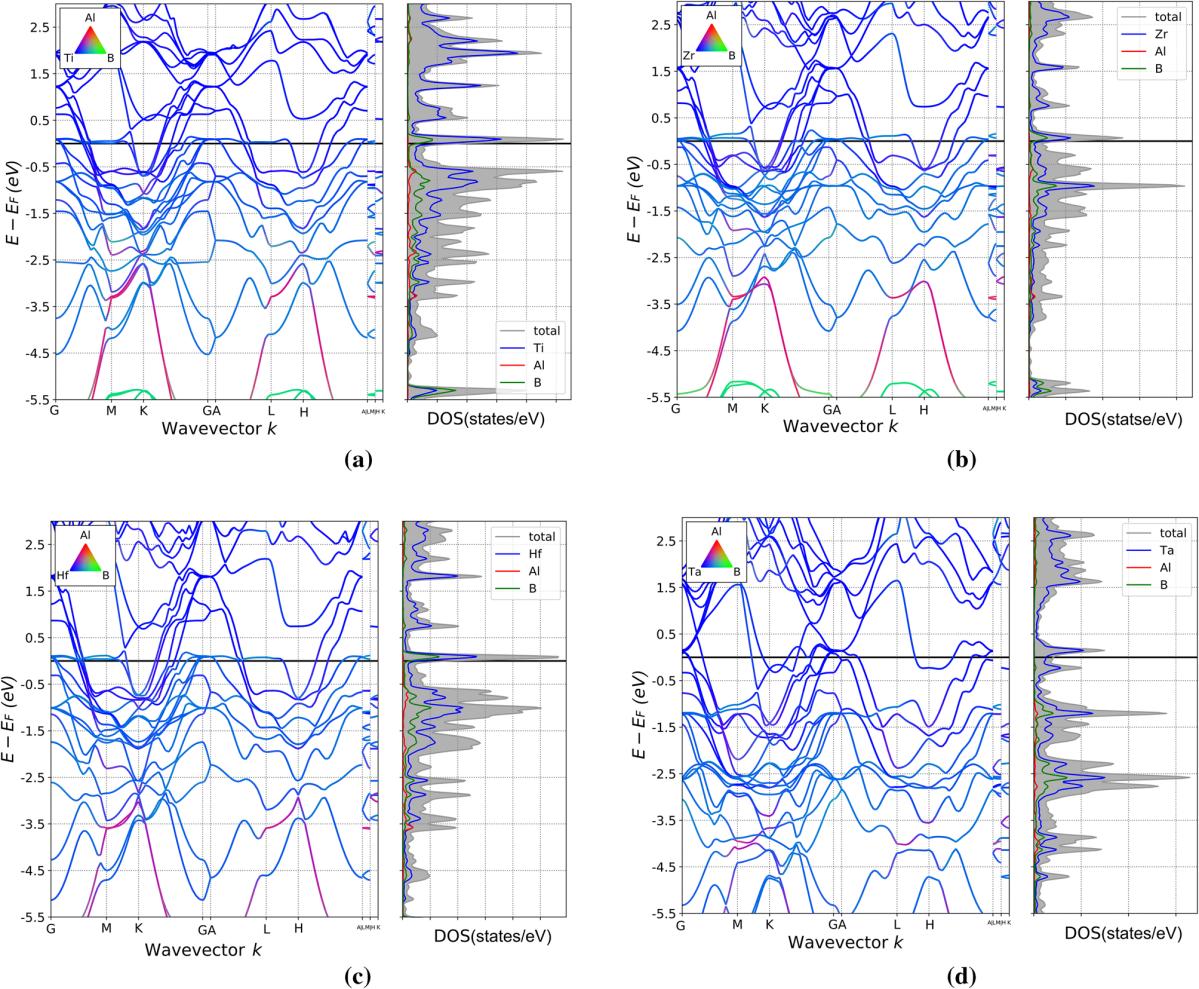
Projected band structures and density of states for Ti_3_AlB_2_ (**a**), Zr_3_AlB_2_ (**b**), Hf_3_AlB_2_ (**c**), Ta_3_AlB_2_ (**d**).

The projected band structures and density of states near the Fermi energy (E_f_) for Ti_3_AlB_2_, Zr_3_AlB_2_, Hf_3_AlB_2_, and Ta_3_AlB_2_ is depicted in Fig. 2. It is reveal that the energy bands of these compounds intersect the Fermi surface, suggesting that Ti_3_AlB_2_, Zr_3_AlB_2_, Hf_3_AlB_2_, and Ta_3_AlB_2_ exhibit electrical conductivity^9^. This has typical characteristics of MAX phase. It can be seen from the energy band that Ti, Zr, Hf, and Ta are dominant in Ti_3_AlB_2_, Zr_3_AlB_2_, Hf_3_AlB_2_, and Ta_3_AlB_2_ materials near the Fermi level. It is also evident that the d orbitals of Ti, Zr, and Hf predominantly occupy the lower portion of the conduction band and the upper portion of the valence band. Al has a minor contribution at the top of the valence band, while it has a very small impact at the bottom of the conduction band, which is almost negligible. B also makes a specific contribution at the bottom of the conduction band and the top of the valence band, primarily in the p orbital. The Fermi level in Ta_3_AlB_2_ is predominantly occupied by the d orbital of the Ta element, while the contribution of Al and B near the Fermi level of Ta_3_AlB_2_ is minimal and negligible.

It can be observed from these figures that the fundamental characteristics in the density of states for the same series of compounds, Ti_3_AlB_2_, Zr_3_AlB_2_, Hf_3_AlB_2_, and Ta_3_AlB_2_, are very similar. Since it is far from the Al atom, the d orbitals of M atom primarily hybridize with the B-p state, and there is some hybrid state with Al atoms. Thus, the M atom forms a covalent bond with the B atom. At the same time, these compounds contain weak M–M covalent bonds. The hybrid state is in a high-energy range, the M–M bond is weaker than the M–B bond.

**Figure 3:**
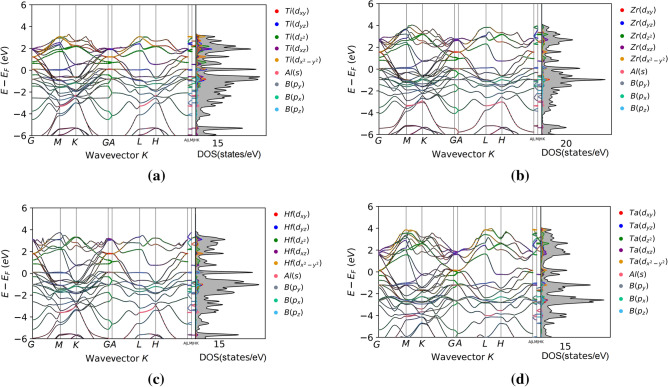
Orbital-weighted band structures and projected density of states of Ti_3_AlB_2_ (**a**), Zr_3_AlB_2_ (**b**), Hf_3_AlB_2_ (**c**), and Ta_3_AlB_2_ (**d**).

Combined with Fig. 3, it can be observed that the lower energy band primarily originates from the B-s state. The spikes in this range are primarily caused by the strong hybridization between the M-d state and the B-p state, specifically the formation of the M–B covalent bond. The energy band at the top of the valence band is the result of hybridization between the M-d state and the Al-p state, which corresponds to M–Al covalent bond. The energy range corresponding to the M–B bond is significantly lower than that of the M–M bond. And, M–B, M–Al bond is stronger than M–M bond.

As shown in Fig. 3, we find that all the d orbitals (d_xy_, d_xz_, d_yz_, d_x_^2^_-y_^2^, and d_z_^2^) in Ti near the Fermi surface of Ti_3_AlB_2_ are given from the band and projected density of states of Ti_3_AlB_2_, Zr_3_AlB_2_, Hf_3_AlB_2_, and Ta_3_AlB_2_. And the contribution of Ti(d_yz_) and Ti(d_x_^2^_-y_^2^) is particularly pronounced. The contribution of Al is very small here, and only p_z_ of B contributes to the density of states. All the d orbitals (d_xy_, d_xz_, d_yz_, d_x_^2^_-y_^2^, and d_z_^2^) in Zr near the Fermi surface of Zr_3_AlB_2_ contribute. Among these, Zr(d_yz_) and Ti(d_x_^2^_-y_^2^) contribute more significantly, while Al contributes very little. Additionally, only the p_z_ orbital of B contributes here. All the d orbitals (d_xy_, d_xz_, d_yz_, d_x_^2^_-y_^2^, and d_z_^2^) in Hf near the Fermi surface of Hf_3_AlB_2_ contribute. Among these, Hf(d_yz_) and Ti(d_x_^2^_-y_^2^) contribute more significantly, while Al contributes very little. B has p_y_ and p_z_ orbitals that contribute, with a larger contribution. All the d orbitals (d_xy_, d_xz_, d_yz_, d_x_^2^_-y_^2^, and d_z_^2^) in Ta near the Fermi surface of Ta_3_AlB_2_ contribute, with Ta(d_x_^2^_-y_^2^), Ta(d_z_^2^), and Ta(d_xz_) making the most significant contributions, while Al and B contribute minimally.

### Lattice vibration

**Figure 4:**
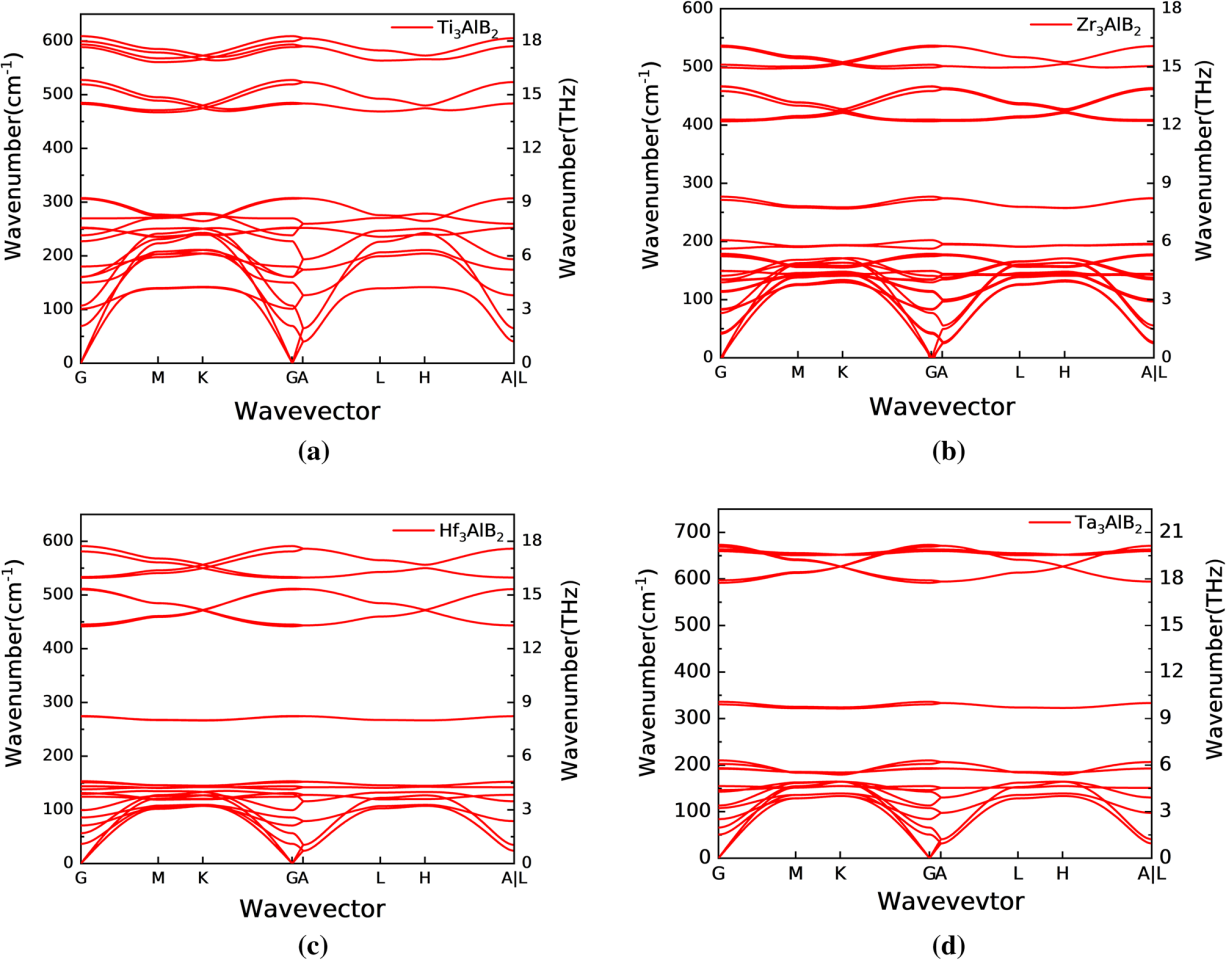
Phonon dispersion of Ti_3_AlB_2_ (**a**), Zr_3_AlB_2_ (**b**), Hf_3_AlB_2_ (**c**), Ta_3_AlB_2_ (**d**).

The lattice vibration spectra of Ti_3_AlB_2_, Zr_3_AlB_2_, Hf_3_AlB_2_, and Ta_3_AlB_2_ are depicted along the direction of high symmetry points in the Brillouin zone as shown in Fig. 4. Firstly, there is no phonon vibration with negative frequency in the phonon dispersion in the studied compounds, which indicates that there is no imaginary frequency and Ti_3_AlB_2_, Zr_3_AlB_2_, Hf_3_AlB_2_, and Ta_3_AlB_2_ are stable.

**Figure 5:**
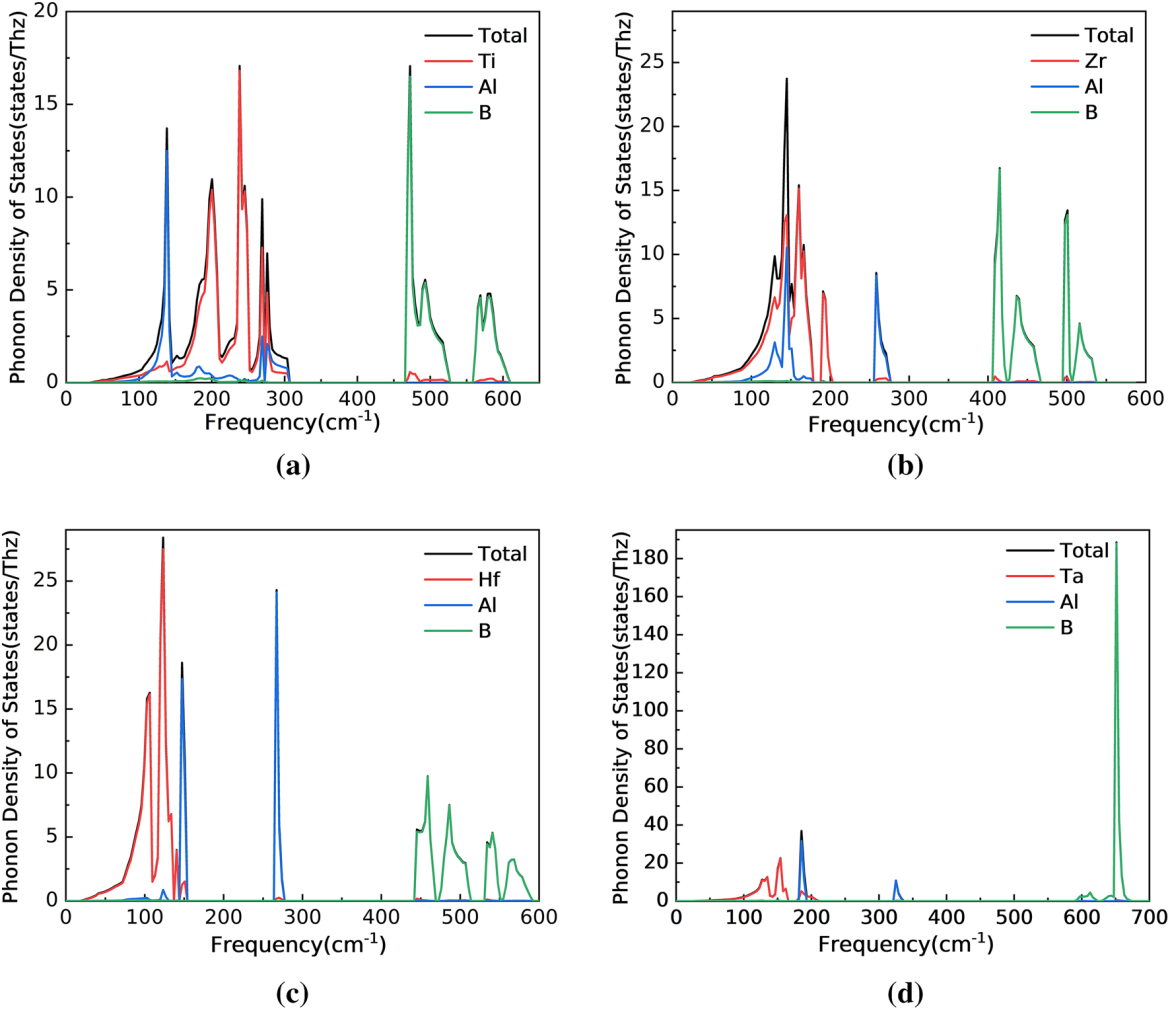
Phonon density of states of Ti_3_AlB_2_ (**a**), Zr_3_AlB_2_ (**b**), Hf_3_AlB_2_ (**c**), and Ta_3_AlB_2_ (**d**).

Secondly, it can be observed from these figures that Ti_3_AlB_2_, Zr_3_AlB_2_, Hf_3_AlB_2_, and Ta_3_AlB_2_ compounds have phonon band gaps. For Ti_3_AlB_2_, the phonon band gap is 306.97 cm^−1^–465.33 cm^−1^. To Zr_3_AlB_2_, the phonon band gap is 276.67 cm^−1^–406.63 cm^−1^. And there are phonon band gaps of 277.33 cm^−1^–441.66 cm^−1^ and 340.66 cm^−1^–589.00 cm^−1^ for Hf_3_AlB_2_, and Ta_3_AlB_2_ respectively. Furthermore, the phonon vibrations of M and Al are below the phonon band gap, while the phonon vibrations above belong to B atoms. Similar to the electronic state, the flat band in the phonon spectrum corresponds to the peak in the phonon state density as shown in Fig. 5. This indicates that the lattice vibration exhibits clear localization characteristics. The frequencies of the optical branches near the long-wave (Γ point) are significantly different, indicating a noticeable ionic character in these compounds.

Thirdly, compared to M and Al atoms, the mass of B atoms is significantly lower. More importantly, B atoms are connected to the lattice through very strong M–B bonds. This results in the vibration frequency of the B atom being significantly higher than that of other atoms. The phonon energy intervals corresponding to M and Al atoms are more complex. On the one hand, based on the electronic structure and chemical bonding mentioned above, M atoms are connected to the lattice through strong M–B bonds and weak M–Al bonds. Therefore, the binding of M atoms to the lattice should be stronger than that of Al atoms. On the other hand, the M atom is much heavier than the Al atom. These factors result in the phonon states corresponding to M and Al atoms being at lower energy levels, but the relative energy level changes of the two are more complicated. And the B atoms are at higher energy levels and form phonon band gaps with them.

In the M_3_AlB_2_ compound, the phonon density of states (PHDOS) below the phonon band gap primarily corresponds to the lattice vibration dynamics of M and Al atoms, while above the band gap, it relates to the lattice vibration of B atoms. There is a sharp peak in the middle in the PHDOS of Zr_3_AlB_2_, Hf_3_AlB_2_, and Ta_3_AlB_2_ due to the vibration of Al atoms. And the proportion of Ti, Zr, Hf, and Ta in PHDOS of Ti_3_AlB_2_, Zr_3_AlB_2_, Hf_3_AlB_2_, and Ta_3_AlB_2_ respectively, increases at low frequencies.

It is assumed that an increase in the number of d electron layers results in and its atom mass, leading to a decrease in the lattice vibration frequency for M atom. For Al and B atoms, although the changing trend of the phonon frequency of the Al atom is similar to that of the M atom, the decisive factor should be the bond strength of M–Al, and the changing trend of the phonon frequency of the B atom is determined by the bond strength of M–B. Among the M_3_AlB_2_ phase compounds, M–Al bond exhibits the lowest chemical bond stiffness in each system, with a stiffness value approximately 1/3–1/2 of the corresponding M-B bond stiffness. Additionally, the M-B bond closest to the Al atom demonstrates the highest stiffness. The results in Fig. 5 also indirectly indicate the changing trend of M–Al bond and M–B bond strength.

### IR and Raman spectra

Theoretically, Ti_3_AlB_2_, Zr_3_AlB_2_, Hf_3_AlB_2_, and Ta_3_AlB_2_ belongs to point group D_6h_ and has 36 vibration modes at the center of the Brillouin zone respectively. The irreducible representation of their vibration modes^73–75^ at the Γ point is in Eq. (9):

9 $${\text{M}} = {\text{2A}}_{{{\text{1g}}}} + {\text{ 3B}}_{{{\text{1u}}}} + {\text{3B}}_{{{\text{2g}}}} + {\text{4A}}_{{{\text{2u}}}} + {\text{ 3E}}_{{{\text{2u}}}} + {\text{ 3E}}_{{{\text{2g}}}} + {\text{ 4E}}_{{{\text{1u}}}} + {\text{ 2E}}_{{{\text{1g}}}}$$

The acoustic branch has two irreducible representation. E_1u_ is doubly degenerate, and the acoustic branch contains 3 lattice waves. And the acoustic branch is as following Eq. (10):

10 $$\Gamma_{{{\text{acoustic}}}} = {\text{ A}}_{{{\text{2u}}}} + {\text{E}}_{{{\text{1u}}}}$$

There are 22 types of irreducible representation in the optical branch, among which E_1g_, E_2g_, E_1u_, and E_2u_ are doubly degenerate, corresponding to 33 lattice waves. The optical branch is as following Eq. (11):

11 $$\Gamma_{{{\text{optic}}}} = {\text{ 2A}}_{{{\text{1g}}}} + {\text{ 3B}}_{{{\text{1u}}}} + {\text{ 3B}}_{{{\text{2g}}}} + {\text{3A}}_{{{\text{2u}}}} + {\text{ 3E}}_{{{\text{2u}}}} + {\text{ 3E}}_{{{\text{2g}}}} + {\text{ 3E}}_{{{\text{1u}}}} + {\text{ 2E}}_{{{\text{1g}}}}$$

Therefore, there are a total of 36 lattice waves. And Table 2 presents the frequencies of phonon vibration modes at the center of the Brillouin zones for Ti_3_AlB_2_, Zr_3_AlB_2_, Hf_3_AlB_2_, and Ta_3_AlB_2_. E_1g_, E_2g_, E_1u_, and E_2u_ are doubly degenerate, and the frequencies of the corresponding phonon vibration modes are identical.

**Table 2:** Vibration mode frequency (Symbol: ω, unit: cm^−1^) and Mulliken symbols of Ti_3_AlB_2_, Zr_3_AlB_2_, Hf_3_AlB_2_, and Ta_3_AlB_2_ at Brillouin zone center.

No	Ti_3_AlB_2_	ω	Zr_3_AlB_2_	ω	Hf_3_AlB_2_	ω	Ta_3_AlB_2_	ω
1	E_2u_	69.4	E_2u_	47.1	E_2u_	36.7	E_2u_	50.8
2	E_2g_	101.3	B_1u_	73.2	B_1u_	56.4	B_1u_	65.8
3	B_1u_	107.3	E_2g_	87.5	E_2g_	70.8	E_2g_	83.9
4	E_1u_	150.2	E_1g_	115.0	E_1g_	85.7	E_1g_	107.8
5	B_2g_	160.6	B_2g_	128.6	B_2g_	99.4	B_2g_	113.2
6	E_1g_	161.0	E_1u_	138.8	E_1u_	124.4	A_1g_	142.9
7	E_2g_	180.1	A_1g_	139.7	A_1g_	129.5	E_1u_	146.4
8	A_1g_	227.2	E_2g_	153.8	E_2u_	131.2	E_2u_	154.9
9	A_2u_	238.2	E_2u_	175.0	A_2u_	138.3	E_2g_	192.2
10	E_2u_	251.5	E_1u_	178.7	B_1u_	144.0	E_1u_	193.3
11	E_1u_	252.5	A_2u_	187.6	E_2g_	151.2	A_2u_	202.6
12	B_1u_	269.8	B_1u_	201.7	E_1u_	153.3	B_1u_	210.2
13	A_2u_	306.3	A_2u_	271.4	A_2u_	274.0	B_2g_	330.5
14	B_2g_	307.6	B_2g_	277.3	B_2g_	274.6	A_2u_	336.1
15	E_1u_	482.7	E_1u_	405.9	E_1u_	441.7	E_1u_	591.7
16	E_2u_	484.9	E_2u_	410.8	E_2u_	445.1	A_2u_	596.9
17	E_2g_	519.2	E_1g_	459.4	E_1g_	510.3	E_1u_	659.7
18	E_1g_	527.4	E_2g_	467.1	E_2g_	511.7	E_2u_	659.9
19	A_2u_	588.6	B_1u_	498.8	A_2u_	531.9	E_1g_	662.7
20	B_1u_	593.5	A_2u_	503.7	B_1u_	533.6	E_2g_	663.7
21	B_2g_	599.5	A_1g_	534.4	B_2g_	581.1	B_2g_	669.2
22	A_1g_	609.0	B_2g_	536.5	A_1g_	591.1	A_1g_	673.2

As shown in Table 2, the highest phonon frequency of Ti_3_AlB_2_ is approximately 609.0 cm^−1^, while Zr_3_AlB_2_ has a highest phonon frequency of about 536.5 cm^−1^. Hf_3_AlB_2_ exhibits a highest phonon frequency of around 591.1 cm^−1^, and Ta_3_AlB_2_ has the highest phonon frequency at approximately 673.2 cm^−1^. Ti_3_AlB_2_, Zr_3_AlB_2_, Hf_3_AlB_2_, and Ta_3_AlB_2_. Although the atom mass of Ti, Zr, Hf, and Ta increases in turn, the highest phonon frequency may be related to atom mass, bond length, and bond angle. Therefore, the highest phonon frequency of Ti_3_AlB_2_, Zr_3_AlB_2_, Hf_3_AlB_2_, and Ta_3_AlB_2_ does not increase completely in accordance with the atom mass of Ti, Zr, Hf, and Ta. It can be seen from the table that for similar crystal structures (Ti_3_AlB_2_, Zr_3_AlB_2_, Hf_3_AlB_2_, and Ta_3_AlB_2_), the Mulliken symbol order corresponding to the characteristic table is not necessarily the same. This discrepancy may be attributed to the distinct nature of the Ti, Zr, Hf, and Ta elements, leading to differences in the fine structure of the crystal. This may be one of the reasons why people use Raman spectroscopy as a “fingerprint spectrum” to distinguish between different crystals and even very similar crystal fine structures.

**Figure 6:**
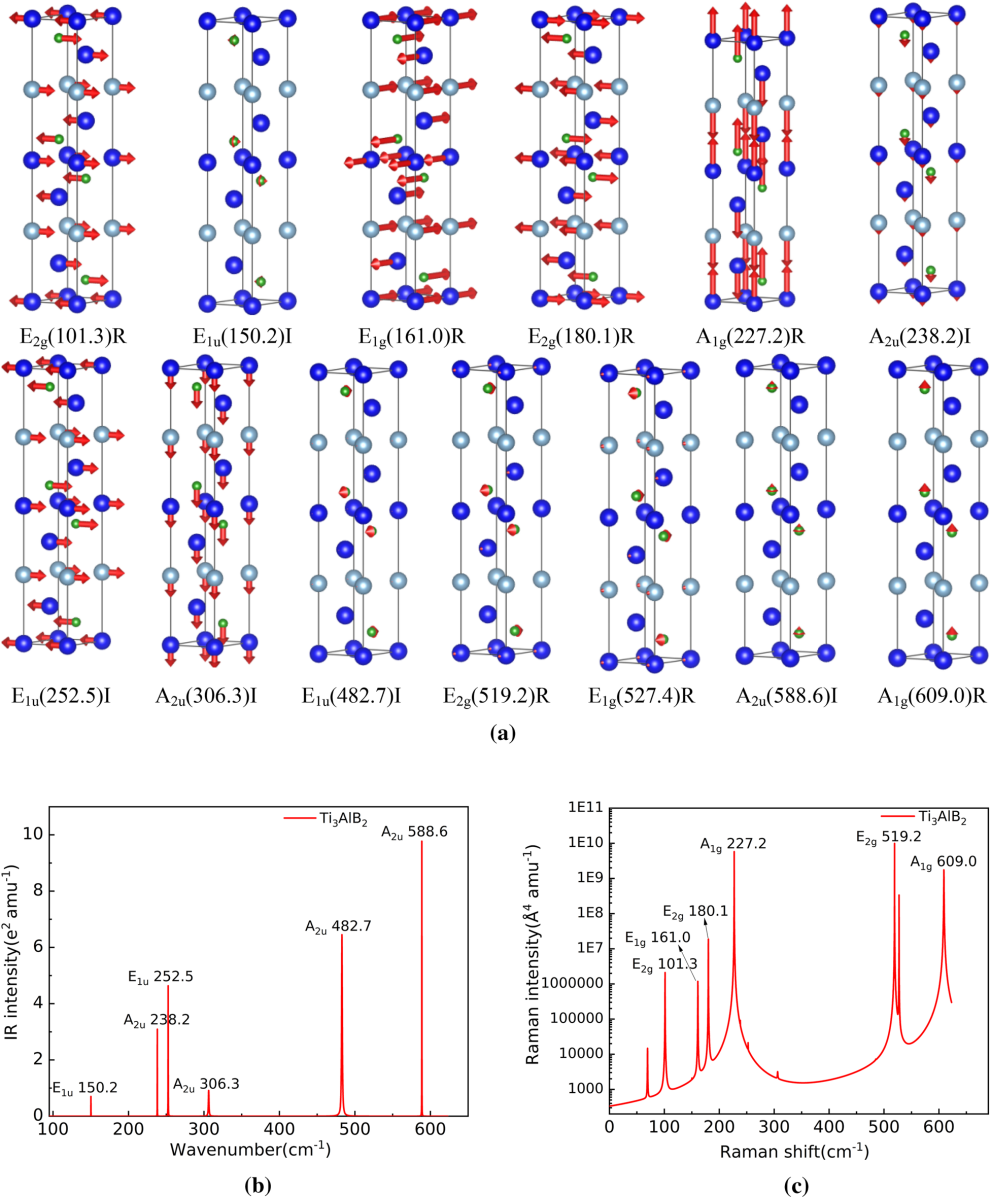
Atom vibration diagram (**a**) (‘I’ represents Infrared activity, ‘R’ represents Raman activity), Infrared and Raman spectra of Ti_3_AlB_2_ (**b** and **c**).

**Figure 7:**
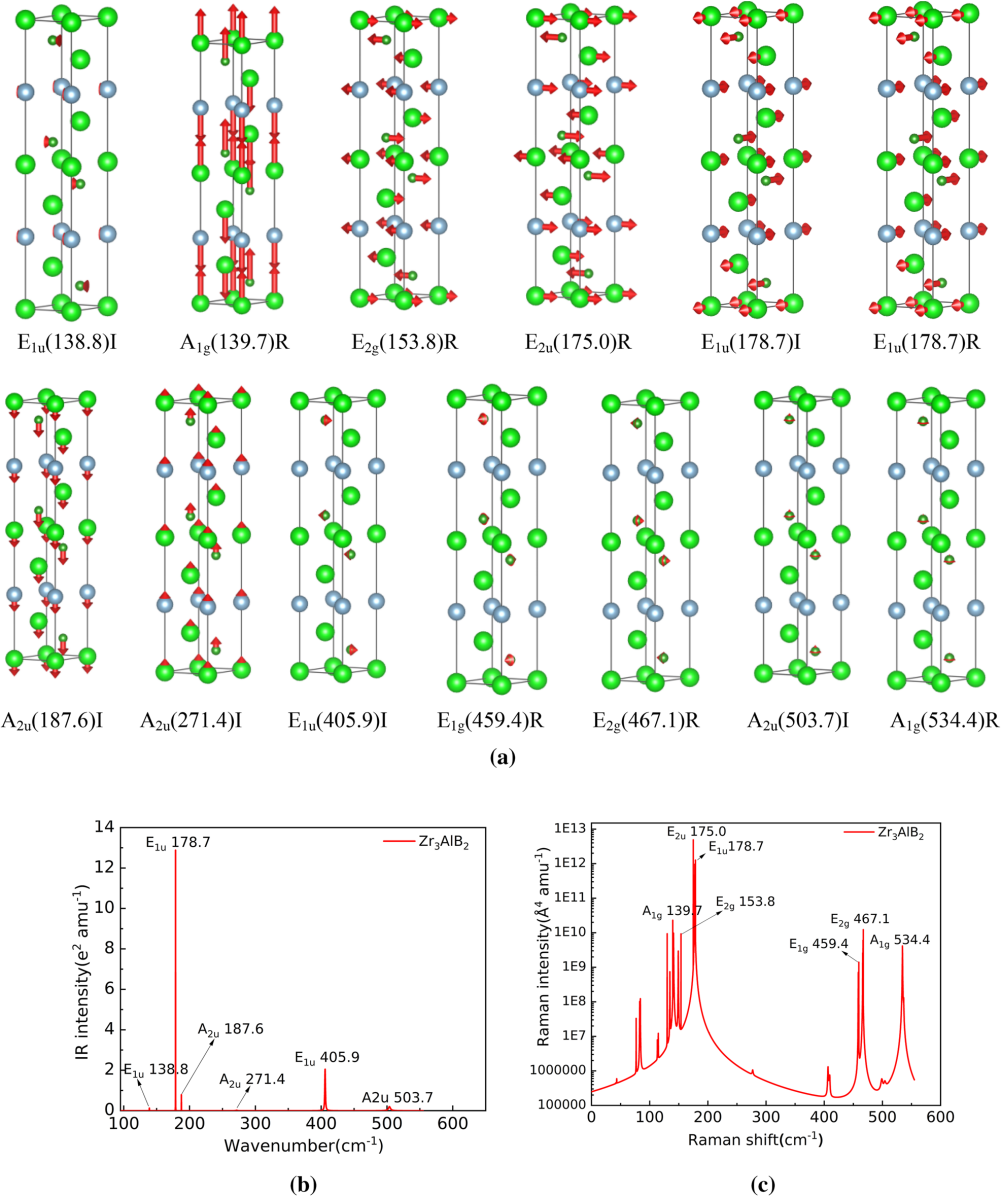
Atom vibration diagram (**a**), Infrared and Raman spectra of Zr_3_AlB_2_ (**b** and **c**).

**Figure 8:**
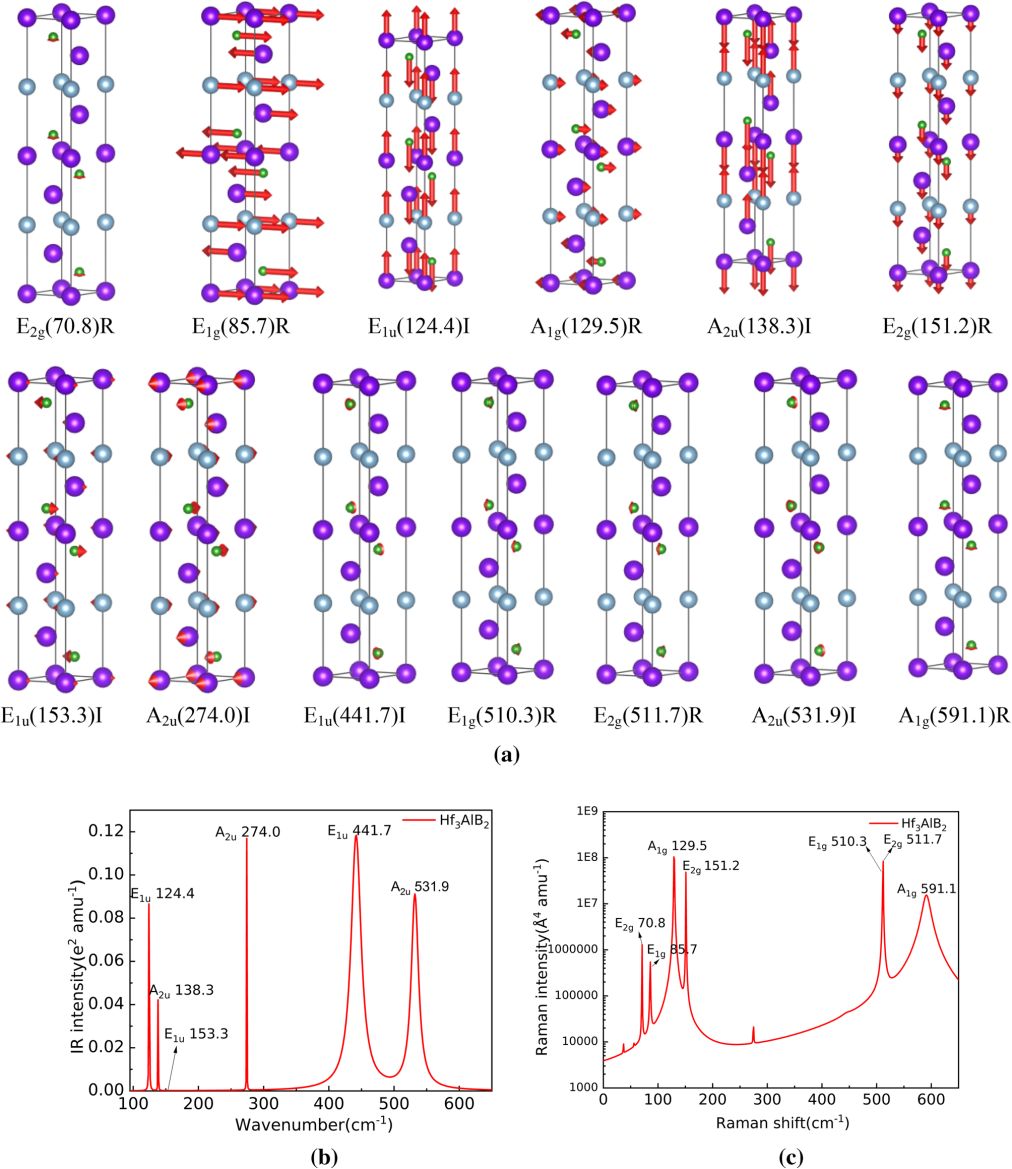
Atom vibration diagram (**a**), Infrared and Raman spectra of Hf_3_AlB_2_ (**b** and **c**).

**Figure 9:**
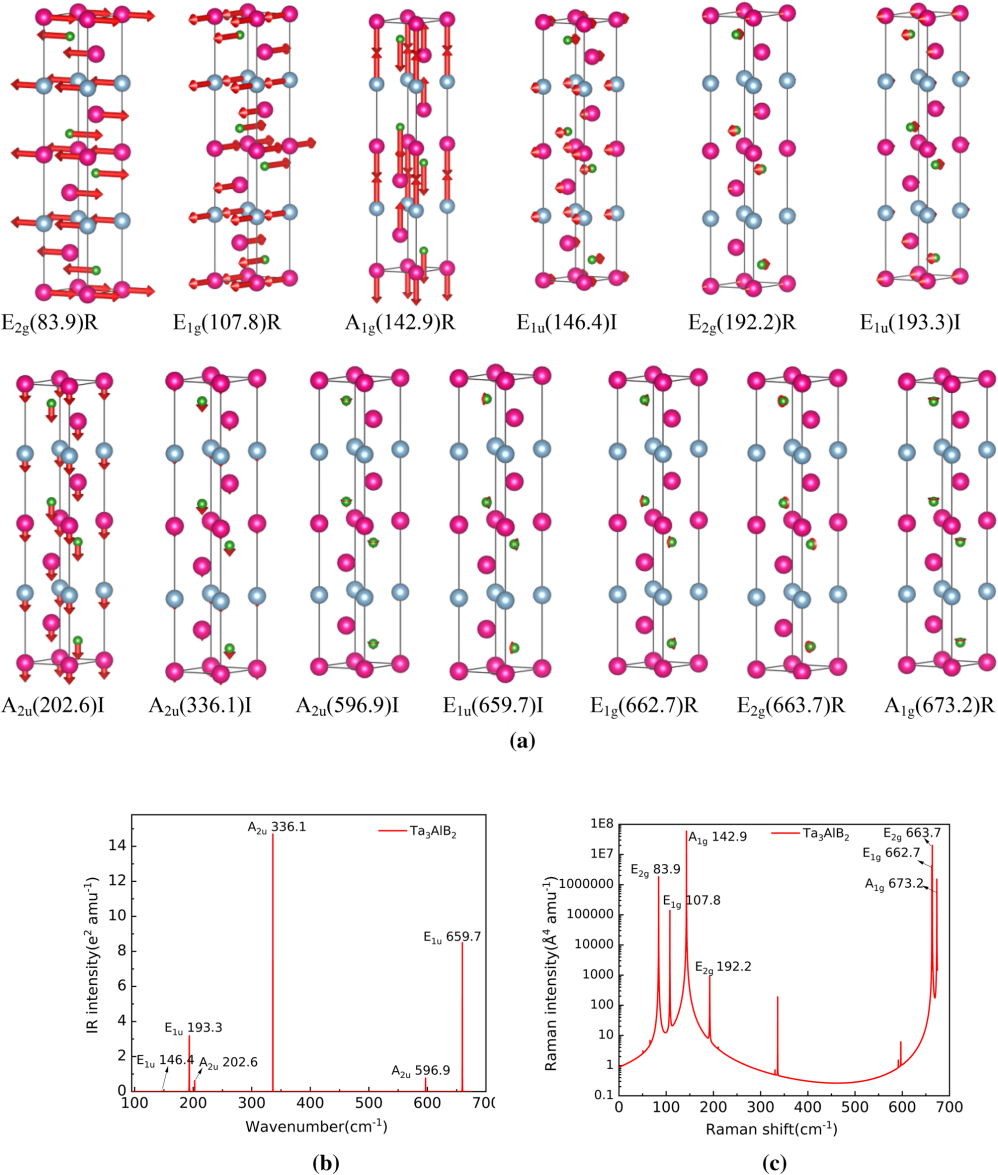
Atom vibration diagram (**a**), Infrared and Raman spectra of Ta_3_AlB_2_ (**b** and **c**).

According to the character table of the point group, A_2u_ and E_1u_ are Infrared-active modes in the optical properties of Ti_3_AlB_2_, Zr_3_AlB_2_, Hf_3_AlB_2_, and Ta_3_AlB_2_, totaling 6 modes: 3A_2u_ + 3E_1u_. The Raman-active modes include A_1g_, E_2g,_ and E_1g_, totaling 7 modes: 2A_1g_ + 3E_2g_ + 2E_1g_. Based on structural optimization, we calculated the atom vibration modes, as well as the Infrared and Raman spectra of Ti_3_AlB_2_, Zr_3_AlB_2_, Hf_3_AlB_2_, and Ta_3_AlB_2_ materials, as depicted in Figs 6, 7, 8, and 9.

The atom vibration diagrams of the Infrared and Raman modes in the Ti_3_AlB_2_ crystal are depicted in Fig. 6a. It can be seen from Fig. 6a that Ti_3_AlB_2_ has 6 Infrared modes: E_1u_(150.2)I, A_2u_(238.2)I, E_1u_(252.5)I, A_2u_(306.3)I, E_1u_(482.7)I, A_2u_(588.6)I. In addition to E_1u_(150.2)I, the low-frequency Infrared vibration is primarily attributed to the vibration of Ti and Al atoms. In contrast, the high-frequency vibration is primarily attributed to the vibration of B atoms. At the same time, the B atom participates in all Infrared vibrations.

E_1u_(150.2)I corresponds to the anti-direction bending vibration in the basal plane of the B atom, E_1u_(482.7)I corresponds to the anti-direction shear vibration in the basal plane of the B atom, and A_2u_(588.6)I corresponds to the longitudinal vibration in the same direction in the basal plane of the B atom. The A_2u_(238.2)I and A_2u_(306.3)I modes represent the longitudinal vibration of Ti, Al, and B atoms along the c-axis direction, while the E_1u_(252.5)I mode corresponds to the shear vibration of Ti, Al, and B atoms along the c-axis direction. Because titanium Ti and Al atoms are relatively heavy, the frequency of these three vibration modes is always low. The E_1u_(482.7)I and A_2u_(588.6)I modes correspond to the vibration of the B atom, respectively. The mass of the B atom is lower, resulting in a higher corresponding frequency.

It can also be seen that Ti_3_AlB_2_ has 7 Raman active modes: E_2g_(101.3)R, E_1g_(161.0)R, E_2g_(180.1)R, A_1g_(227.2)R, E_2g_(519.2)R, E_1g_(527.4)R, A_1g_(609.0)R from Fig. 6a. It is evident that the atom motion associated with the Raman vibration mode primarily involves elongation and bending vibration of covalent bond chains of M–Al–B. Among them, the vibrations of Ti and Al atoms commonly result in the E_2g_(101.3)R, E_1g_(161.0)R, E_2g_(180.1)R, and A_1g_(227.2)R vibrations at low frequency. The lower frequency is due to the greater mass of Ti and Al atoms and the deformation of the weaker M–Al bond during its bending vibration. At high frequencies, the E_2g_(519.2)R, E_1g_(527.4)R, and A_1g_(609.0)R modes are mainly affected by the vibration of B atoms, with the vibration of Ti and Al playing a minor role. This could be attributed to the strong M–B bond and the relatively low mass of the B atom, which results in higher vibration frequencies in these stretching vibration modes. These findings are consistent with the phonon density of states diagram Fig. 5a. The frequencies E_2g_(101.3)R, E_1g_(161.0)R, E_2g_(180.1)R, E_2g_(519.2)R, and E_1g_(527.4)R indicate that atoms in the Ti_3_AlB_2_ crystal vibrate in opposite directions perpendicular to the c axis. On the other hand, A_1g_(227.2)R and A_1g_(609.0)R show that the atoms in the Ti_3_AlB_2_ crystal vibrate in opposite or same directions along the c axis, respectively. Figure 6b shows the position and vibration intensity of the specific peak of the Infrared active vibration mode. Figure 6b illustrates that the A_2u_(238.2)I, E_1u_(252.5)I, E_1u_(482.7)I, A_2u_(588.6)I modes in the Ti_3_AlB_2_ structure exhibit strong Infrared vibration intensity, while the E_1u_(150.2)I and A_2u_(306.3)I modes have lower Infrared vibration intensity. And Fig. 6c provides their specific peak positions and intensities. The higher vibration intensities can be observed in the Raman vibration modes of Ti_3_AlB_2_, including E_2g_(101.3)R, E_1g_(161.0)R, E_2g_(180.1)R, A_1g_(227.2)R, E_2g_(519.2)R, E_1g_(527.4)R, and A_1g_(609.0)R, as shown in Fig. 6c.

The atom vibration of Infrared and Raman modes are shown in Fig. 7a. Zr_3_AlB_2_ has 6 Infrared modes: E_1u_(138.8)I, E_1u_(178.7)I, A_2u_(187.6)I, A_2u_(271.4)I, E_1u_(405.9)I, A_2u_(503.7)I. The B atom also participates in all the Infrared vibrations. In addition to E_1u_(138.8)I, the low-frequency Infrared vibration is dominated by the vibration of Zr and Al atoms, while the high-frequency vibration is dominated by the vibration of B atoms. E_1u_(138.8)I corresponds to the anti-direction bending vibration in the basal plane of B atom, E_1u_(405.9)I corresponds to the anti-direction shear vibration in the basal plane of B atom, and A_2u_(503.7)I corresponds to the longitudinal vibration in the same direction in the basal plane of B atom. A_2u_(187.6)I, A_2u_(271.4)I are the longitudinal vibration of Ti, Al and B atoms along the c-axis direction, and E_1u_(178.7)I is the shear vibration of Zr, Al and B atoms along the c-axis direction. Due to the relatively heavy Zr and Al atoms, the frequency of these three vibration modes is always not high. E_1u_(405.9)I and A_2u_(503.7)I correspond to the vibration of B atom, respectively. The mass of B atom is lower, so the corresponding frequency is higher.

It can also be seen from Fig. 7a that Zr_3_AlB_2_ has 7 Raman active modes: A_1g_(139.7)R, E_2g_(153.8)R, E_2u_(175.0)R, E_1u_(178.7)R, E_1g_(459.4)R, E_2g_(467.1)R and A_1g_(534.4)R. Among them, the modes of A_1g_(139.7)R, E_2g_(153.8)R, E_2u_(175.0)R, E_1u_(178.7)R at low frequencies are the common results of Zr and Al atom vibrations. The E_1g_(459.4)R, E_2g_(467.1)R and A_1g_(534.4)R modes at high frequencies are dominated by the vibration of B atoms, and the vibration of Zr and Al is almost negligible. This may be due to the higher strength of the M-B bond and the significantly smaller mass of the B atom, which makes these stretching vibration modes have higher vibration frequencies. These results correspond to the phonon density of states Fig. 5b. E_2g_(153.8)R, E_2u_(175.0)R, E_1u_(178.7)R, E_1g_(459.4)R, E_2g_(467.1)R show that the atoms in Zr_3_AlB_2_ crystal vibrate in opposite directions perpendicular to the c axis, while A_1g_(139.7)R, A_1g_(534.4)R show that the atoms in Zr_3_AlB_2_ crystal vibrate in opposite and same directions along the c axis, respectively.

Figure 7b,c shows specific peak positions and intensities of Infrared and Raman spectra in Zr_3_AlB_2_. Figure 7b shows that E_1u_(178.7)I, A_2u_(187.6)I, and E_1u_(405.9)I in the Ti_3_AlB_2_ structure have strong Infrared vibration intensity. The Infrared vibration intensity of A_2u_(187.6)I is low, and the Infrared vibration intensity of E_1u_(138.8)I, A_2u_(271.4)I, and A_2u_(503.7)I is very low, which may be difficult to observe experimentally. While, it can be seen from Fig. 7c that in the Raman vibration mode of Zr_3_AlB_2_, A_1g_(139.7)R, E_2g_(153.8)R, E_2u_(175.0)R, E_1u_(178.7)R, E_1g_(459.4)R, E_2g_(467.1)R, A_1g_(534.4)R have higher vibration intensity, but A_1g_(139.7)R, E_2g_(153.8)R, E_1u_(178.7)R peaks are annihilated between other peaks, which may be difficult to identify experimentally. And it is interesting that the E_1u_(178.7)peak has both Infrared activity and Raman activity.

The schematic diagram of atom vibration, Infrared and Raman spectra of Hf_3_AlB_2_ are given in Fig. 8a,b,c, respectively. It can be seen from Fig. 8a that Hf_3_AlB_2_ has 6 Infrared modes: E_1u_(124.4)I, A_2u_(138.3)I, E_1u_(153.3)I, A_2u_(274.0)I, E_1u_(441.7)I, A_2u_(531.9)I. Figure 8b shows the position and vibration intensity of the specific peak of the Infrared active vibration mode. E_1u_(124.4)I, A_2u_(138.3)I, A_2u_(274.0)I is clear in the Hf_3_AlB_2_ Infrared spectra in Fig. 8b. E_1u_(441.7)I, A_2u_(531.9)I have strong Infrared vibration intensity and larger broadening. However, the Infrared vibration intensity of E_1u_(153.3)I is very low and not observable in the study. It can also be seen from Fig. 8a that Ti_3_AlB_2_ has 7 Raman active modes: E_2g_(70.8)R, E_1g_(85.7)R, A_1g_(129.5)R, E_2g_(151.2)R, E_1g_(510.3)R, E_2g_(511.7)R, A_1g_(591.1)R. Figure 8c gives their specific peak positions and intensities. It can be seen from Fig. 8c that the Raman vibration mode of Hf_3_AlB_2_, Hf_3_AlB_2_ has E_2g_(70.8)R, E_1g_(85.7)R, A_1g_(129.5)R, E_2g_(151.2)R, E_1g_(510.3)R, E_2g_(511.7)R, and A_1g_(591.1)R all have high vibration intensity. And the broadening of A_1g_(591.1)R peak is larger. While, E_1g_(510.3)R E_2g_(511.7)R peak is very close and almost unrecognizable.

The vibration diagram of atoms, Infrared and Raman spectra of Ta_3_AlB_2_ are shown in Fig. 9a,b,c, respectively. From Fig. 9a, we can see that Ta_3_AlB_2_ also has 6 Infrared modes: E_1u_(146.4)I, E_1u_(193.3)I, A_2u_(202.6)I, A_2u_(336.1)I, A_2u_(596.9)I, E_1u_(659.7)I. Figure 9b shows the position and vibration intensity of the specific peak of the Infrared active vibration mode. E_1u_(193.3)I, A_2u_(336.1)I, E_1u_(659.7)I in Hf_3_AlB_2_ structure has strong Infrared vibration intensity. A_2u_(202.6)I, A_2u_(596.9)I have lower Infrared vibration intensity, and the Infrared vibration intensity of E_1u_(146.4)I is very low and almost difficult to identify. From Fig. 9a, it can also be seen that Ti_3_AlB_2_ has 7 Raman active modes: E_2g_(83.9)R, E_1g_(107.8)R, A_1g_(142.9)R, E_2g_(192.2)R, E_1g_(662.7)R, E_2g_(663.7)R, A_1g_(673.2)R. Figure 9c gives their specific peak position and intensity. From Fig. 9c, it can be seen that all of peaks of Raman spectra in Ta_3_AlB_2_ have high vibration intensity, while E_1g_(662.7)R, E_2g_(663.7)R is very close and need to be carefully distinguished in the experiment.

## Conclusion

In summary, the study provided detailed insights into the structural, electronic, and mechanical properties of M_3_AlB_2_ compounds using first-principles calculations. The results contribute to the understanding of these materials and their potential applications in various fields. The findings can guide further experimental investigations and the design of new materials with tailored properties. Combined with the calculation results of the intensity and distribution of Infrared and Raman vibrations of M_3_AlB_2_, it can be seen that the Infrared and Raman vibrations of M and Al atoms account for a large proportion at low frequencies, while the vibration of lighter B atoms accounts for a large proportion at high frequencies. Some of the Infrared or Raman peaks of Zr_3_AlB_2_, Hf_3_AlB_2_, and Ta_3_AlB_2_ are difficult to distinguish, probably because the frequencies of the two vibration modes are very close, or because the weak Infrared or Raman peaks are easily annihilated in the substrate spectral lines and are difficult to identify. This paper predicts that the crystal Infrared and Raman spectra of Ti_3_AlB_2_, Zr_3_AlB_2_, Hf_3_AlB_2_, and Ta_3_AlB_2_ provide a good theoretical basis for future experiments, but the hyper-Raman spectrum and the change of Raman characteristic spectrum under stress have the potential for further research.

## Data Availability

All data included in this study are available upon request by contact with the corresponding author Shengzhao Wang (shengzw@nyist.edu.cn).
